# Infection of dogs by *Leishmania infantum* elicits a general response of IgG subclasses

**DOI:** 10.1038/s41598-020-75569-6

**Published:** 2020-11-02

**Authors:** A. I. Olías-Molero, I. Moreno, M. J. Corral, M. D. Jiménez-Antón, M. J. Day, M. Domínguez, J. M. Alunda

**Affiliations:** 1 Department of Animal Health, Faculty of Veterinary Medicine, Universidad Complutense, Madrid, Spain; 2 Unidad de Inmunología Microbiana, Instituto de Salud Carlos III, Carretera de Majadahonda-Pozuelo, Km.2.2, Majadahonda, Madrid, Spain; 3 School of Veterinary and Life Sciences, Murdoch University, Murdoch, WA Australia

**Keywords:** Parasitic infection, Parasite host response, Applied immunology, Infection

## Abstract

*Leishmania infantum* is the etiological agent of zoonotic visceral leishmaniasis. In endemic areas, canine infections are considered the main source of infection for human populations. Therefore, any control of human leishmaniasis must include the control of canine infections. Chemotherapy of leishmaniasis is inadequate and canine immunoprophylaxis has important limitations. Reports on the response of infected dogs are abundant but no clear picture of immune events has emerged. To shed some light on these shortcomings the specific IgG subclass response was followed in 20 Beagle dogs experimentally infected with *L. infantum* using monoclonal antibodies (MAb) specific for canine IgG_1_, IgG_2_, IgG_3_ and IgG_4_, along with ELISA and flow cytometry. Results showed that parasitic infection elicits a general response of all IgG subclasses, with a predominant IgG_1_ response and without any evidence of IgG_1_/IgG_2_ dichotomy. These findings suggest that the inconsistent results reported previously could be related to the lack of specific reagents and not to the actual differences in the immune response of infected animals. Differential IgG subclass reactivity in ELISA and cytometry and the analysis of the reacting antigens could facilitate the diagnosis and prognosis of the disease and provide a useful tool for adequate therapeutics and vaccine development against leishmaniasis.

## Introduction

Visceral leishmaniasis (VL) is a vector-borne parasitic disease caused by the infection with protozoa of the genus *Leishmania*. The disease is widely distributed and it is considered the second most lethal parasitic disease in humans, after malaria^1,2^. Apparent expansion of VL has been reported^3,4^ and some factors have been incriminated such as new epidemiological patterns, extensive human travel, and climate change, among others^5^. Two species are mainly responsible for human visceral infections: *L. donovani* and *L. infantum* (= *L. chagasi*). There are some reports on *L. donovani* infections in non-human hosts although its transmission cycle is mainly anthroponotic. However, *L. infantum*, prevalent in Asia, South America and southern Europe, also affects dogs, which are considered the main reservoir for human infections (zoonotic VL, ZVL)^1,6,7^. Canine leishmaniasis affects animals of both sexes, all ages and breeds and the clinical course is generally chronic. Thus, *L. infantum* constitutes an important issue in both public health and veterinary medicine. In humans the highest prevalence and worst prognosis of ZVL are found in children, elderly and individuals with non-efficient immune system (e.g. HIV+ and other concurrent infections, immunodepressed recipients of solid organ transplants^8,9^ or systemic lupus erythematosus–SLE-patients). In areas where ZVL is endemic, human infections by *L. infantum* are not very frequent, but the prevalence of canine infections in Europe can occasionally reach over 30%^10^; and in South America there are millions of dogs currently infected. Consequently, any control measure to limit the extension of human disease must necessarily include the control of canine leishmaniasis. Unfortunately, chemotherapy has serious shortcomings (e.g. low efficacy, high price of drug presentations of reduced toxicity)^11^, environmental prophylaxis is impracticable and available veterinary vaccines need substantial improvements^12^.

Immune regulation of leishmaniasis has been extensively analyzed^13^ in mouse models of VL. Susceptibility has been associated with a Th2 polarized response with production of IgG_1_ and IgE, whereas resistant mouse lines are able to mount a Th1-based response with production of specific IgG_2a_^14^. The Th1/Th2 framework, with the dichotomy leading to the control or proliferation of *Leishmania*, although relatively well characterized in mice, with susceptible and refractory strains, is clearly an oversimplification, and fundamental roles of Treg and some cytokines have been described in the tuning of *Leishmania*-host interface^15^. The immune response in mice to *L. infantum* (or *L. donovani*) has little predictive value as to the response in humans, in which the Th1/Th2 polarization is far from clear^16^. In the case of *L. infantum*, dogs are also natural hosts for the trypanosomatid and the interest on the response elicited, both for diagnosis, prognosis or vaccination, has fueled research. Consequently, many reports on the cellular and humoral response and the cell types and cytokines involved have been published. Generally, resistance in canine leishmaniasis has been associated to upregulation of the Th1-cytokines (IFN-γ, TNF-α) with a significant cellular response; on the contrary, progression of the disease would be linked to a Th2-biased response (e.g. IL-4) with high production of IgG^17^. It is well established that leishmaniasis in dogs is characterized by the limited cellular response and the high levels of antibodies (Ab), particularly IgG^18–23^. However, reports on the IgG subclasses involved and their correlation to the clinical status of the infected animals are confusing. Thus, several studies have linked the active disease to a predominant IgG_1_ response^24–28^ whereas in other reports it has been associated to a significant IgG_2_^29–36^ or a mixed response^20,37–39^ despite using comparable techniques and reagents.

Purification, identification and production of monoclonal antibodies (MAb) against the canine IgG subclasses (IgG_1_, IgG_2_, IgG_3_, IgG_4_)^40–42^ has allowed their comparison with marketed secondary antibodies (anti dog IgG_1_ and IgG_2_). Their use in the study on several dog diseases, including ZVL^43^, has highlighted the potential bias in the published reports on the IgG subclasses in ZVL^44^. Despite the elapsed time since those results were obtained (> 10 years), very few studies using MAb specific for canine IgG subclasses have been published^45–48^, whereas many contributions using commercial nonspecific immunological reagents have been published afterwards^28,33,35^. This is most critical since the inaccurate determination of the immune scenario (cytokines, cells and immunoglobulins, particularly IgG subclasses) in canine resistance, resilience or susceptibility is a strong shortcoming to develop vaccines or to assess the efficacy of chemotherapy or immunotherapy. In our case, the availability of sera from a large number of dogs subjected to an experimental infection with a recently isolated wild strain of *L. infantum*^49^ allowed us to determine the dynamics of production of specific IgG subclasses and their relationship to the clinical course of the experimental animals.

## Results

### Specificity of MAb anti-IgG subclasses

Since no purified canine IgG subclasses were available, specificity of MAb (B6, E5, A3G4, A5) was assessed by capture ELISA. MAb A5 (anti-IgG_4_) reacted only with the serum captured by itself as coating Ab, thus confirming its specificity for dog IgG_4_ (Table 1). Preliminary trials performed with biotinylated MAb E5 (anti-IgG_2_) yielded inconsistent results. Therefore, its determination required the use of peroxidase-labeled anti-mouse IgG_2b_. With this approach E5 reacted with the serum captured by itself besides showing nonspecific recognition of anti-IgG_3_. The cross-reactivity observed was expected since both the revealing labeled Ab (E5, anti-IgG2) and the coating anti-dog IgG_3_ MAb (A3G4) were of mouse IgG_2b_ isotype. Interestingly, A3G4 showed a strong self-recognition (anti-IgG_3_) and lower reactivity with the serum captured by B6 (anti-IgG_1_). MAb B6 (anti-IgG_1_), employed as secondary (detection) Ab, slightly reacted when the coating MAb was itself (anti-IgG_1_) whereas it strongly reacted with dog serum captured by A3G4 (anti-IgG_3_). These results with capture ELISA confirmed that MAb recognized the four canine IgG subclasses. Therefore, they could be used to determine the dynamics of IgG subclasses. However, MAb displayed variable affinities and their value had to be confirmed in the indirect ELISA determinations in the infected dogs along the infection with *L. infantum*.

**Table 1:** Summary of reactivity of monoclonal antibodies (MAb) against canine IgG subclasses in the specificity test carried out with capture ELISA.

Coating MAb	Revealing labeled MAb			
	B6 Anti-IgG_1_ [IgG_1_]	E5 Anti-IgG_2_ [IgG_2b_] + anti-IgG_2b_^a^	A3G4 Anti-IgG_3_ [IgG_2b_]	A5 Anti-IgG_4_ [IgG_1_]
B6	+	–	+ +	–
E5	–	+ + +	–	–
A3G4	+ + +	+ + +	+ + +	–
A5	–	–	–	+ + +

High (+++), moderate (++) and mild (+) reactivity. In brackets, murine isotypes of MAb.

^a^Anti-mouse IgG_2b_ to develop anti-IgG_2_ reaction.

### Experimental *L. infantum* infection in dog elicits the elevation of all IgG subclasses determined by ELISA

**Figure 1:**
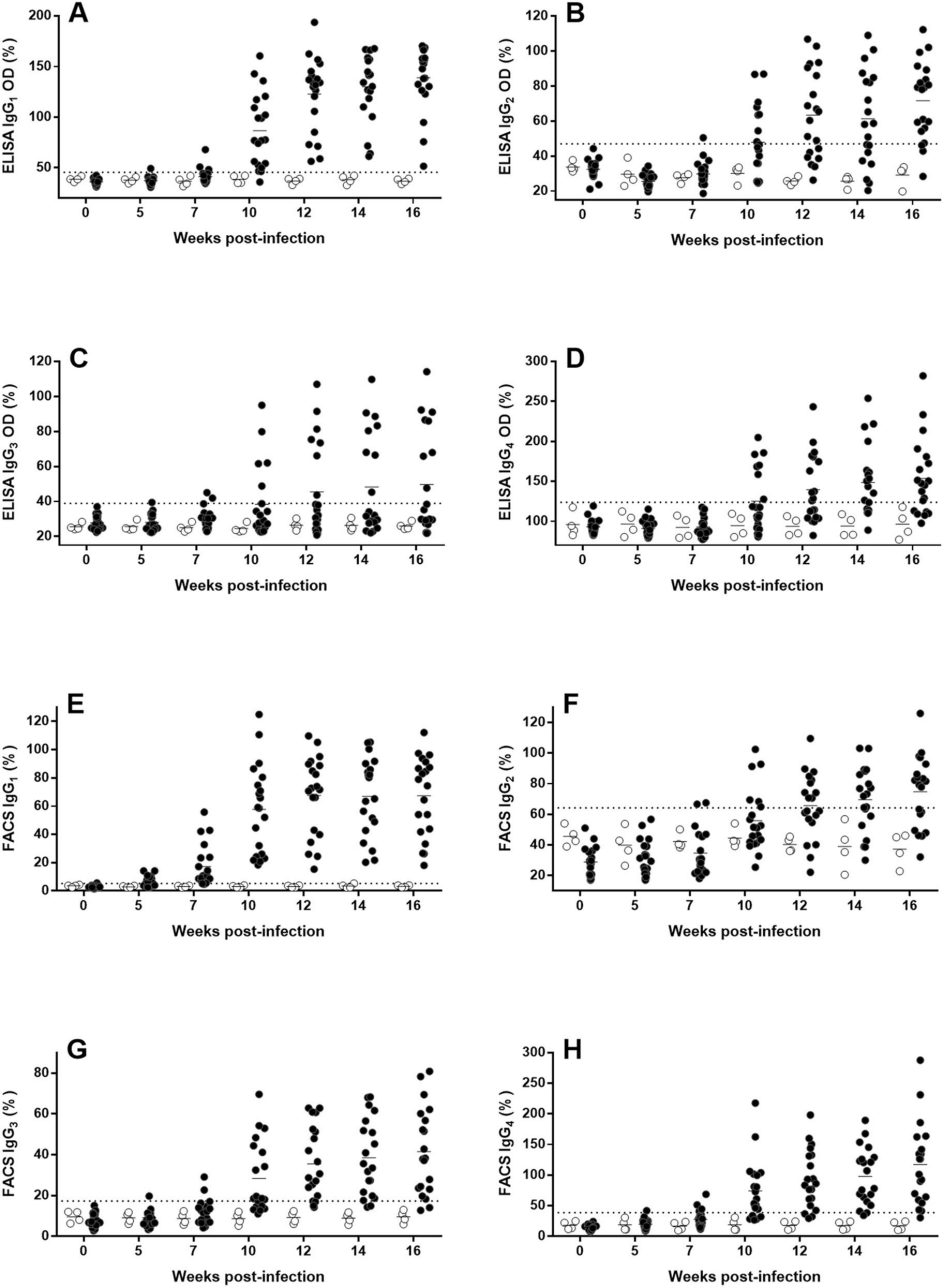
Serum anti-*Leishmania* IgG subclasses of Beagle dogs experimentally infected with *L. infantum* estimated by ELISA and flow cytometry and subclass-specific monoclonal antibodies (MAb). ELISA [(**A**) IgG_1_; (**B**) IgG_2_; (**C**) IgG_3_; (**D**) IgG_4_] assay was carried out with soluble leishmanial antigen and results are expressed as % of the positive control sera. Flow cytometry (FACS) [(**E**) IgG_1_; (**F**) IgG_2_; (**G**) IgG_3_; (**H**) IgG_4_] was performed with whole promastigotes. Solid circles: inoculated animals; empty circles: non-inoculated control dogs. Dotted line: cut-off level (%). Figure was prepared with GraphPad Prism 6.01 software.

**Figure 2:**
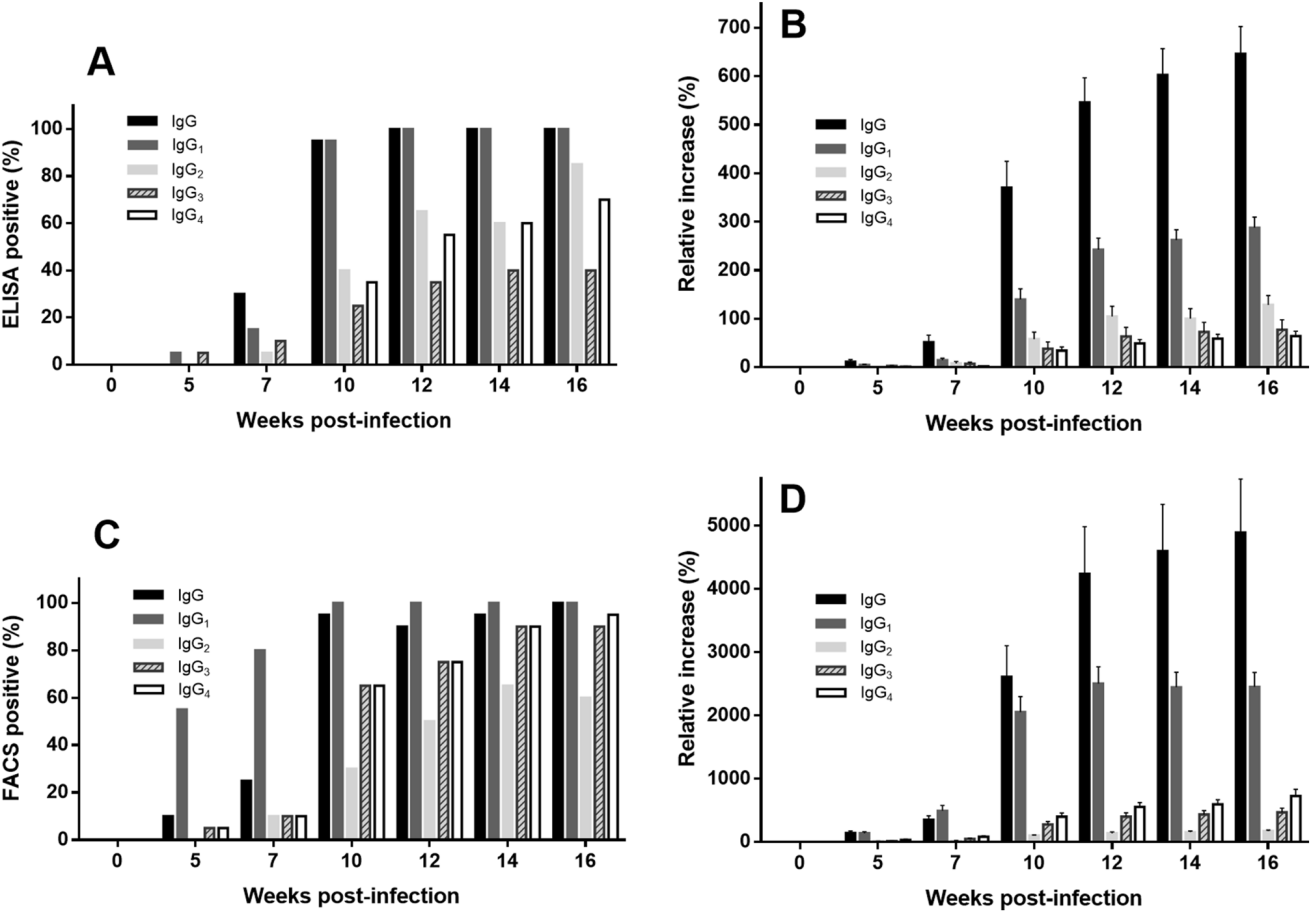
Positive dogs (above cut-off) (% of inoculated animals) and relative increase of anti-*Leishmania* serum IgG subclasses (% over pre-infection levels) of Beagle dogs experimentally infected with *L. infantum*. (**A**) Dogs positive by ELISA (IgG, IgG_1_, IgG_2_, IgG_3_ and IgG_4_); (**B**) Relative increase of specific IgG and IgG subclasses; (**C**) Dogs positive by flow cytometry (FACS) (IgG, IgG_1_, IgG_2_, IgG_3_ and IgG_4_); (**D**) Relative increase of specific IgG and IgG subclasses. ELISA was performed with leishmanial soluble antigen and FACS was carried out with whole promastigotes. Values of antibody increase by ELISA (**B**) and FACS (**D**) are mean ± standard error. Figure was prepared with GraphPad Prism 6.01 software.

ELISA results indicated that inoculated Beagles showed an increase of all anti-*L. infantum* IgG subclasses related to the infection while the uninfected control dogs remained under the cut-off value during the experimental period. However, increase over pre-infection levels varied among IgG subclasses (Fig. 1A–D). Thus, while on week 7 post infection (pi), 30% (6/20) of the animals were already positive with total IgG, IgG_1_, the most abundant subclass was only elevated in 3/20 dogs and IgG_2_ and IgG_3_ levels were over the cut-off value in 5% and 10% of the animals, respectively. Specific IgG_4_ response was delayed and it was detected only from week 10 pi (7/20, 35%) onwards. At this sampling time (week 10 pi) 95% of the dogs (19/20) were IgG and IgG_1_ positive. Considering all the serum samples only 14 out of 20 infected dogs showed detectable levels of anti-*Leishmania* IgG_4,_ and less than half of the animals displayed positive IgG_3_ reactions (40%, 8/20) (Fig. 2A). All dogs, except #24, were anti-*Leishmania* IgG_2_ positive at some sampling along the infection.

Average values of total IgG and IgG subclasses increased in a time-dependent manner despite the individual variations (Fig. 2B). Specific anti-*Leishmania* IgG levels strongly correlated with IgG_1_ (r = 0.9438), IgG_2_ (r = 0.8558) and IgG_4_ (r = 0.7152) (*P* < 0.0001) but not with IgG_3_ (r = 0.2961; *P* = 0.0010). Accordingly, whereas IgG_1_, IgG_2_ and IgG_4_ were highly correlated (r = 0.7155–0.8441; *P* < 0.0001) no clear relationship emerged with IgG_3_ (r = 0.250–0.269; *P* < 0.01). Considering the cumulative Ab response, estimated by the trapezoidal method (AUC), IgG levels up to week 16 pi strongly correlated with IgG_1_ and IgG_2_ but not with the other subclasses. IgG_2_ correlated with IgG_1_ and IgG_4_ but not with IgG_3_ (Table 2).

**Table 2:** Correlation (Spearman *ρ* value) between the cumulative Ab response of IgG subclasses along the experimental infection of dogs (16 weeks) with *L. infantum*.

	IgG	IgG_1_	IgG_2_	IgG_3_	IgG_4_
IgG	–	0.8511****	0.6150**	ns	ns
IgG_1_	0.8511****	–	0.7008***	ns	ns
IgG_2_	0.6150**	0.7008***	–	ns	0.6271**
IgG_3_	ns	ns	ns	–	ns
IgG_4_	ns	ns	0.6271**	ns	–

**P < 0.01; ***P < 0.001; ****P < 0.0001; ns: not significant.

Given the wide variation found in the immune response, levels of Ab were compared to the clinical score (CS) of the experimental dogs (Supporting Information Table 1). No correlation was found between Ab response and the clinical status in the early phases of the infection (5 weeks pi). However, IgG_1_ and IgG_2_ were positively correlated with the value of CS of experimental Beagle dogs on week 10 pi, and IgG_1_ also on week 16th. IgG_2_:IgG_1_ and IgG_3_:IgG_1_ ratios did not significantly vary with the clinical status of the animals but the IgG_4_:IgG_1_ ratio showed a negative correlation with CS from week 10 pi onwards (Table 3).

**Table 3:** Correlation (Spearman *ρ* value) between serum concentration of IgG subclasses and ratios, and the clinical score (CS) of dogs infected with *L. infantum*, at different pi times.

Time	IgG_1_	IgG_2_	IgG_3_	IgG_4_	IgG_2_:IgG_1_	IgG_3_:IgG_1_	IgG_4_:IgG_1_
5 wpi	ns	ns	ns	ns	ns	ns	ns
10 wpi	0.6599**	0.7281***	ns	ns	ns	ns	− 0.5227*
14 wpi	ns	ns	ns	ns	ns	ns	− 0.5070*
16 wpi	0.5102*	ns	ns	ns	ns	ns	− 0.5163*

wpi: weeks post inoculation. ns: not significant. *P < 0.05; **P < 0.01; ***P < 0.001.

### Flow cytometry determination of specific IgG subclasses against *L. infantum* in experimentally infected dogs

MAb allowed the detection of the four IgG subclasses produced against *L. infantum* using flow cytometry. All inoculated animals were IgG and IgG_1_ positive (over cut-off value) at some moment of the infection whereas six dogs did not show any detectable specific IgG_2_ and in one dog no specific IgG_3_ and IgG_4_ were found (Fig. 1E–H). FACS-estimated response against *L. infantum* showed a steady time-dependent increase of specific IgG subclasses, besides total IgG. An early response of IgG_1_ was found and by week 5 pi 55% (11/20) of the infected Beagles were positive, whereas response of other subclasses was low (IgG_3_ and IgG_4_, 5% of the animals) or absent (IgG_2_) in this sampling time (Fig. 2C). This late response of IgG_2_ was also evident by week 7 pi, when 80% of the inoculated dogs were positive to IgG_1_ and only 2 out of 20 animals had IgG_2_ levels over the cut-off value. Contrary to findings in ELISA, relative increase of IgG_4_ was higher than that from IgG_3_ and IgG_2_ (Fig. 2D). Moreover, in a similar way to that found with ELISA, the widest variation among inoculated dogs was observed in specific IgG_4_ response; values in the last sampling of some dogs experienced > 200% increase. Concentration of IgG subclasses determined by flow cytometry were correlated among them and with total IgG (r = 0.8245–0.9405; *P* < 0.0001) but no relationship between their levels and the CS of the dogs was observed. FACS values were correlated with ELISA values, particularly for total IgG (r = 0.8427; *P* < 0.0001) and IgG_1_ (r = 0.8399; *P* < 0.0001). Other subclasses also showed significant correlations (*P* < 0.0001) but with lower “r” values (IgG_2_: 0.7234; IgG_4_: 0.7001; IgG_3_: 0.6161).

## Discussion

Inoculation of age- and sex- matched Beagle dogs with amastigotes of a fresh canine isolate of *L. infantum* elicited, under our conditions, an infection in all of them, developing clinical signs and lesions compatible with leishmaniasis. Progression of the disease was accompanied by a steady increase of all specific IgG subclasses determined (IgG_1_, IgG_2_, IgG_3_, IgG_4_). These results do not support the polarized framework described in natural and experimental canine leishmaniasis with either a predominant IgG_1_^24–28^ or IgG_2_^29–35,50^ response in clinically affected dogs.

Several causes can account for the inconsistent findings on anti-*Leishmania* specific IgG_1_ and IgG_2_ response in ZVL, including the general absence of quantitative determinations of IgG and IgG subclasses and the variable experimental designs (e.g. infective dose, number of animals, epidemiological surveys), methodologies (e.g. ELISA, Western blot, antigen and antigen preparation). For sure these factors must play important roles but, in our view, at least two main reasons have hampered the determination of the relative value of IgG_1_:IgG_2_ and its diagnostic and prognostic potential in ZVL. The first relates to the scarce available information on the real role of IgG subclasses in dogs and their relationship to that found in mice^14,44,51^ or hamsters^52–54^. The second, is linked to the lack of specificity of immunological determinations which, in turn, are dependent on the specificity of the reagents. Mazza and coworkers characterized and developed MAb against canine IgG subclasses^40–42^. In our case purified canine IgG subclasses were not available, and the specificity of the MAb obtained from the hybridoma lines (B6: anti-IgG_1_; E5: anti-IgG_2_; A3G4: anti-IgG_3_; A5: anti-IgG_4_) could not be tested directly but rather by capture ELISA. A5 and E5 showed a good recognition of IgG_4_ and IgG_2_, respectively. The cross reactivity found in capture ELISA between IgG_2_ and IgG_3_ was expected as anti-mouse IgG_2b_ was employed for the amplification to detect E5 (anti-IgG_2_, IgG_2b_). Cross reactivity between IgG_1_ and IgG_3_ with MAb B6, already reported, could be due to a prozone phenomenon or competition for epitopes. Serum IgG_1_ concentration in healthy dogs is over 20 fold that of IgG_3_ (8.17 ± 0.95 mg/mL vs. 0.36 ± 0.43 mg/mL), and quantitative assays suggested that there was no simultaneous reactivity^41^. We have not performed quantitative ELISA but no correlation (ρ) between the IgG_1_ and IgG_3_ response against *L. infantum* was observed in our study (see below). Thus, despite some shortcomings, the available MAb could be used to evaluate canine IgG subclasses along the experimental infection.

Our results show that the concentrations of all IgG subclasses were increased along the experimental infection with *L. infantum* in a time-dependent manner. This elevation of the four subclasses confirms and extends the results obtained with the same MAb by other authors^43,45–48^. Response was variable among experimental dogs. IgG_1_ and IgG_2_, the most abundant subclasses, were also the dominant ones reacting in ELISA with leishmanial soluble antigen (SLA). Up regulation of specific IgG_1_, IgG_4_ and IgG_3_, compared to IgG_2_, has been reported in naturally infected dogs^43,46^. In our case, all inoculated animals developed clinical signs, lesions and biopathological alterations compatible with leishmaniasis and *L. infantum* infection was confirmed by biopsy. ELISA results showed that the highest relative increase over pre-infection values corresponded to specific IgG_1_ (over 100% after 10 weeks of infection) followed by IgG_2_ > IgG_3_ > IgG_4_ (Fig. 2B). The early increase of IgG_1_ (95% positive on week 10 pi) (Fig. 2A) suggests that, using ELISA and SLA as antigen, it could be a serum marker of active (recent) *L. infantum* infections whereas that of IgG_2_ would indicate a more advanced infection. The IgG_4_:IgG_1_ ratio was a good marker of the clinical course although the limited functional characterization of canine IgG subclasses^51^ and the lack of stoichiometry in our indirect ELISA preclude any speculation before more studies are performed.

The determination of IgG response to *L. infantum* using flow cytometry is less common, although some studies have been carried out^55^. Given the lack of subclass specificity of marketed Ab, the apparently different response found in vaccinated dogs with Leishvaccine (IgG_1_) or Leishmune (IgG_2_) must be taken cautiously^56^. Our results with FACS showed a general pi time-related increase of all subclasses and ELISA and flow cytometry values were correlated. This supports the value of the MAb for both assays and the consistency of the immune response, although IgG_1_ was detected earlier by cytometry. Earlier IgG_1_ response detected by FACS could be compatible with the recognition of membrane-bound antigen (Ag) and hence its potential value as infection marker. Interestingly, no correlation was found, at any sampling point, between the clinical score (CS) and IgG subclasses pattern. Contrarily to the ELISA results, the relative increase of IgG_4_ along the follow-up was higher than that of IgG_2_ and IgG_3_ (Fig. 2D); IgG_4_ values of experimental dogs (12 wpi) were also higher (*ca.* two fold) than those determined in dogs with chronic infection. Results seem factual and the possible significance (significance in acute phase, chronic phase) of the high relative levels of anti-*Leishmania* IgG_4_ requires further research. The differential reactivity found in ELISA and FACS points towards variable sets of Ag being exposed and detected by the techniques and their degree of denaturalization (SLA vs. promastigotes). Scarce reactivity of IgG_2_ with FACS, compared to ELISA, suggests that target Ag for IgG_2_ response should be intracellular (soluble/cytosolic). Further analyses with individual Ag (purified, WB) could confirm this hypothesis.

Results found in the follow-up of experimentally infected dogs with *L. infantum* support that the mouse-based Th1 (IgG_2a_: resistance)/Th2 (IgG_1_: susceptibility) framework does not represent the actual events in canine leishmaniasis. Marketed anti-IgG_1_ and anti-IgG_2_ are not specific of dog IgG subclasses^43,44,48^ and it is very likely that variable and inconsistent results obtained with them^28,33,35,57^ could be biased.

Our study was carried out under controlled conditions and dogs were exposed to a single infection whereas under field conditions polyparasitism (along with other pathogens) is the rule. Moreover, animals were genetically close, the infective dose was very high, with amastigotes inoculated by IV route. The experimental design was efficient achieving well established infections but these factors could affect the immune response elicited, particularly in the early phases of parasite dissemination. Therefore, further research is needed to confirm our findings in naturally-acquired infections and dog breeds.

Despite these limitations, we can conclude that MAb employed allowed the detection of specific canine IgG subclasses against *L. infantum* by ELISA or cytometry. Cross reactivity found between MAb recognizing IgG_1_ and IgG_3_ in capture ELISA possibly is not highly significant given their differential abundance in serum (*ca.* 20 fold) and the different patterns found for both subclasses along the experimental infection. The concentrations of all subclasses were increased after inoculation and IgG_1_ was the predominant Ab and also the earliest to appear^45^. Differential subclasses pattern found with ELISA and FACS and the lack of correlation between CS and FACS-determined response could be related to the different antigen sets and epitopes detected. Careful analyses of these Ag could give significant clues on the role of IgG subclasses in infected animals. This is critical to get an accurate picture of the immune events in ZVL and could facilitate the diagnosis and prognosis of the disease as well as provide a useful tool for better therapeutics and vaccine development.

## Material and methods

### *Leishmania infantum* strain

*L. infantum* strain was obtained from the spleen of a naturally infected dog from southern Spain (Órgiva, Granada), clinically and serologically diagnosed. Details on the isolation of parasites to inoculate the dogs have been previously published^49^. Briefly, the spleen was cut into small pieces and homogenized in a glass-in-glass tissue grinder. Suspensions were centrifuged and cell pellets were treated with cell lysis buffer. Isolation was performed under sterile conditions and amastigotes were kept at 4 °C for 24 h and used to inoculate dogs. The isolate was characterized (MCAN/ES/2016/Granada-UCM) using published kinetoplast primers and by a specific PCR-hybridization-ELISA with a cloned 196 bp of *L. infantum* kDNA^58,59^.

### Experimental infection of dogs with *L. infantum* and follow-up

Dogs (24 female Beagles) were obtained when they were 4–5 months old (Envigo, France) and housed at the animal facility Nr ES280790000091 of the Faculty of Veterinary Medicine UCM (Madrid). Details of the housing, inoculation and follow-up of the experimental animals have been previously published^49^. Briefly, when the animals reached 10–11 months age, 20 were inoculated (cephalic vein) with 10^8^ amastigotes of *L. infantum*/animal, in 1 mL. Four animals were kept as uninfected control dogs. Blood samples were obtained before infection and on weeks 5, 7, 10, 12, 14 and 16 post inoculation (pi) to perform biopathological and immunological determinations. Dogs were observed daily and subjected to complete clinical examination by a veterinarian blinded to the experimental design. Follow up included IFAT and infection status of the animals on week 16th pi by popliteal lymph node biopsy. Clinical scoring (CS) of the animals (Suplementary Information) included clinical signs and lesions, and biochemical and hematological abnormalities, and was determined following a previous publication^49^.

### Monoclonal antibodies (MAb)

#### Hybridoma lines

Frozen hybridoma lines secreting mouse MAb against dog IgG_1_ (B6), IgG_2_ (E5), IgG_3_ (A3G4) and IgG_4_ (A5)^40–42^ were supplied by Ms. S. Holt (Veterinary Faculty, Bristol, UK). Hybridoma lines were thawed and cultured at 37 ºC in RPMI-1640 medium (BioWhittaker, Lonza) supplemented with 10% heat inactivated fetal calf serum, 1% l-Glutamine 200 mM (BioWhittaker, Lonza), and 100 U/mL penicillin plus 100 μg/mL streptomycin (BioWhittaker, Lonza). Several subcultures were performed to obtain supernatants.

Isotyping of MAb was carried out by capture ELISA. Briefly, 96-well high binding microtiter plates (NUNC, Thermo Fisher) were coated with 3 μg/mL (50 µL/well) of goat polyclonal anti-mouse IgG (SouthernBiotech) in HCO_3_^−^/CO_3_^−^ (0.1 M pH 9.7), overnight at 4 ºC. After blocking (2% BSA, 75 μL/well, 30 min, 37 ºC) and three washes with PBS-Tween, supernatants from hybridoma cultures (8 replica/hybridoma line) were added (50 μL/well) and incubated at 37 ºC, 2 h. Plates were washed as above and peroxidase labeled antibody (Ab) against murine isotypes (SouthernBiotech) and light chains were added: anti-IgA, anti-IgM, anti-IgG_1_, anti-IgG_2a_, anti-IgG_2b_, anti-IgG_3_, anti-kappa and anti-lambda at 1/2000 dilution and incubated at room temperature (RT) for 30 min. Plates were extensively washed (5×) and color reaction was developed with 100 μL/well OPD (1 mg/mL) in citrate–phosphate buffer (0.1 M citric acid-0.2 M sodium phosphate, pH 5.3) with hydrogen peroxide (1/1000). Reaction was stopped with H_2_SO_4_ 3 N (50 μL) and absorbance read at 492 nm with Varioskan LUX (Thermo Fisher Scientific). It was confirmed that hybridoma lines B6 and A5 secreted IgG_1_-kappa Ab whereas IgG_2b_-kappa Ab were produced by lines E5 and A3G4.

#### Purification of MAb

Prefiltered supernatants (SFCA 0.2 µm, Corning) from hybridoma cultures were immunoadsorbed to a protein column (HiTrap 1 mL Protein G HP, GE Healthcare) with peristaltic pump EP-1 Econo Pump (BioRad) and a flow of 1.5 mL/min. Column was washed (PBS, pH 7.2) and bound Ab were eluted with NH_4_OH 0.5 N. Fractions (*ca.* 0.5 mL) with significant protein concentration (280 nm, spectrophotometer SmartSpec Plus, BioRad) were dialyzed against PBS in a visking tube of 12–14 KDa (Medicell Membranes Ltd) for 24 h, at 4 ºC, three changes, and the final concentration was determined with the RC-DC Protein Assay kit (BioRad).

#### Labeling of MAb and specificity

Purified MAb were labeled with biotin (EZ-Link Sulpho-NHS-LC-Biotin, Thermo Fisher) using manufacturer’s indications with minor modifications. Efficacy of labeling was assessed by indirect ELISA using total dog IgG purified as above to coat microtiter plates (10 µg/mL; 50 µL/well). After blocking (BSA 2%, 30 min, 37 ºC) biotin-labeled MAb were added (50 μL/well) and serially diluted in PBS-BSA-Tween (PBS-BSA-T), 1 h, RT. Reaction was developed with 50 μL/well peroxidase-labeled streptavidin (Streptavidin-HRP, SouthernBiotech) 1/2000 in PBS (30 min, RT) and color development was quantified as above (see isotyping). All MAb were positive, except E5 (anti-IgG_2_) despite re-labeling with double concentration of biotin^60^.

Specificity of MAb was assessed by capture ELISA using 2.5 µg/mL MAb to coat microplates. Serum from a healthy dog was added (1/200, 1 h, 37 ºC) and rabbit serum was used as negative control. Biotin-labeled MAb (B6, A3G4, A5) were used at 1/400, and unlabeled MAb (E5) at 1 µg/mL (1 h, RT). MAb peroxidase-streptavidin (SouthernBiotech), and for E5, peroxidase-labeled anti-mouse IgG_2b_ (SouthernBiotech) (1/2000, 30 min, RT) were used to detect Ab reactivity.

### Anti-*Leishmania* IgG subclasses of experimentally infected dogs determined by ELISA

Procedures to propagate promastigotes by back transformation of amastigotes from the original isolate and to obtain soluble leishmanial antigen (SLA) have been described previously^49^. Briefly, promastigotes were cultured in RPMI 1640 medium, supplemented with antibiotic–antimycotic mixture (BioWhittaker), l-glutamine (BioWhittaker), 10% fetal calf serum (Gibco) and human urine (1%). SLA was obtained by centrifugation of mid-log phase promastigotes subjected to freezing-and-thawing cycles.

Levels of specific IgG and subclasses in the infected dogs were determined by indirect ELISA using as positive control the serum from a dog with an immunologically and parasitologically confirmed chronic natural infection by *L. infantum*; negative control serum came from a healthy dog. Microplates were coated with 10 μg/mL SLA (50 μL/well) overnight at 4 ºC. After blocking (2% BSA, 30 min), dog sera were added (50 μL/well) at 1/400 (total IgG), 1/100 (IgG_1_, IgG_3_ and IgG_4_) or 1/50 (IgG_2_) in PBS-BSA-T (1 h, 37 ºC). Biotinylated secondary Ab anti-IgG_1_ (MAb B6), anti-IgG_3_ (MAb A3G4) and anti-IgG_4_ (MAb A5) were used at 1/500, 1/1000 and 1/1000, respectively, in PBS-BSA-T (50 μL/well) (1 h, RT). Unlabeled MAb (E5, anti-IgG_2_) was used at a concentration of 1 µg/mL and was incubated at 37 ºC. Secondary polyclonal anti-IgG (goat anti-Dog IgG H + L-HRP, Bethyl Laboratories, Cat. No. A40-123P) was used at 1/2000 in PBS-BSA-T (30 min, RT). Biotinylated MAb were detected with HRP-labeled streptavidin (SouthernBiotech), 1/2000 in PBS-BSA-T (50 μL/well, 30 min, RT). For the unlabeled MAb (anti-IgG_2_), peroxidase-labeled anti-mouse (goat anti-Mouse IgG_2b_, Human ads-HRP, SouthernBiotech, Cat. No. 1090-05) was employed using the same conditions. Color was developed and OD read as above. Results were expressed as % of the positive control sera (OD of the sample/OD positive control × 100). Mean % OD + 3 standard deviations—SD—of the pre-infection values was used as the cut-off value. Relative increases (%) over pre-infection values were also estimated.

### Determination of anti-*Leishmania* IgG subclasses by flow cytometry (FACS)

The same negative and positive controls used for ELISA were employed in the evaluation of total IgG and IgG subclasses. Mid-log phase promastigotes of the homologous isolate of *L. infantum* were diluted in PBS (5 × 10^8^cells/mL) and divided in 50 μL aliquots in 1.5 mL Eppendorf tubes. An equal volume of serum was added for each experimental animal and sampling time. Tubes were kept ice-cooled for 1 h and reaction was stopped by adding 1 mL/tube 1% formaldehyde in PBS. Contents of the eppendorf tubes was divided onto 5 tubes (200 μL/tube), replenished with BD FACSFlow buffer and centrifuged (15,000×*g*, 1 min). Cell pellets were resuspended in 100 μL of PBS containing 1 μg/mL from each unlabeled MAb, incubated (1 h, RT), washed with FACSFlow and centrifuged (15,000×*g*, 1 min). Anti-mouse fluorescein labeled Ab (Alexa Fluor 488-conjugated AffiniPure Goat Anti-Mouse IgG, Fcγ Fragment Specific, Jackson ImmunoResearch, Cat. No. 115-545-008) was added at 1/500 (100 μL/tube). To determine total IgG, cells were resuspended with fluorescein labeled anti-IgG (FITC-conjugated AffiniPure Rabbit anti-Dog IgG H + L, Jackson ImmunoResearch, Cat. No. 304-095-003) (100 μL/tube) at 1/100. All tubes were incubated (in the dark, 30 min, RT), washed and their contents transferred onto 5 mL cytometry tubes (Corning Falcon) using FACSFlow buffer to get 0.5–1 mL/tube. Cellular analysis was performed with a flow cytometer BD FACSCalibur (Becton Dickinson) and the programme CellQuest Pro v4.0.2. Fluorescent cells were counted (10,000 events, FL-1 filter) and results, mean fluorescence intensity (MFI), were expressed as above (see ELISA).

### Ethics

The experimental protocol followed the legislation of the European Commission (Directive 63/2010/UE) and Spanish transposition (Royal Decree 53/2013) and the 3Rs guidelines. Design and procedures were approved by the Ethical Committee of the Veterinary Faculty (CEEA VET), UCM committee for animal experimentation and the Comunidad de Madrid Regional Government (Ref. PROEX 329/15). All personnel in direct contact with the animals were officially qualified for animal experimentation (ECC/566/2015).

### Statistical analysis

ELISA values were expressed as percentage (%) of the OD value found for the positive control serum (OD of samples/ average OD of positive control × 100); and as relative increase (%) over pre-infection OD values. FACS values were expressed the same way but employing MFI. Cumulative Ab response was estimated using the trapezoidal method to determine areas under the curve (AUC) of each animal from pre-infection to 16 weeks pi point. Relationship between the different subclass levels or the different techniques, as well as between CS and subclass levels (with different techniques), were evaluated using the non-parametric Spearman correlation. In all statistical analyses, the level of significance was set at *P* < 0.05. Figures and statistical analysis were performed using GraphPad Prism 6.01 software.

## Supplementary information

Supplementary Information.

## Data Availability

All data generated or analyzed during this study are included in this published article.
