# Supplementary material for: Infection of dogs by Leishmania infantum elicits a general response of IgG subclasses
Source: Sci Rep. 2020 Nov 2;10:18826. doi: 10.1038/s41598-020-75569-6 (PMC7606601; doi:10.1038/s41598-020-75569-6)
Supplement: Supplementary file 1 — Supplementary Information. [file 41598_2020_75569_MOESM1_ESM.docx]

**SUPPLEMENTARY MATERIAL**

**Infection of dogs by *Leishmania infantum* elicits a general response of IgG subclasses**

Olías-Molero A. I.^1^, Moreno, I.^2^, Corral, M. J.^1^, Jiménez-Antón, M. D.^1^, Day, M.J.^3^, Domínguez, M.^2^, Alunda, J.M.^1^*

^1^ Department of Animal Health, Faculty of Veterinary Medicine, Universidad Complutense, Madrid, Spain

^2^ Unidad de Inmunología Microbiana, Instituto de Salud Carlos III, Carretera de Majadahonda-Pozuelo, Km.2.2, Majadahonda, Madrid, Spain

^3^ School of Veterinary and Life Sciences, Murdoch University, Murdoch, WA, Australia

*Corresponding author

Email: [jmalunda@ucm.es](mailto:jmalunda@ucm.es) (JMA)

**Table 1 Supplementary Material.**

Individual clinical score (CS) of Beagle dogs experimentally infected with *Leishmania infantum* at different post inoculation times.*

| Dog | Time | | | |
| --- | --- | --- | --- | --- |
|  | 5 wpi | 10 wpi | 14 wpi | 16 wpi |
| #1 | 2 | 11 | 12 | 16 |
| #2 | 1 | 1 | 11 | 15 |
| #3 | 2 | 9 | 11 | 14 |
| #4 | 2 | 3 | 17 | 19 |
| #5 | 3 | 7 | 16 | 14 |
| #6 | 1 | 10 | 9 | 10 |
| #7 | 1 | 12 | 11 | 12 |
| #8 | 1 | 9 | 15 | 17 |
| #9 | 0 | 9 | 15 | 19 |
| #10 | 2 | 4 | 9 | 15 |
| #12 | 2 | 10 | 18 | 17 |
| #13 | 1 | 12 | 18 | 19 |
| #14 | 0 | 8 | 15 | 13 |
| #17 | 2 | 2 | 5 | 7 |
| #18 | 2 | 2 | 5 | 10 |
| #19 | 2 | 1 | 12 | 13 |
| #20 | 1 | 1 | 5 | 4 |
| #21 | 2 | 9 | 11 | 14 |
| #23 | 2 | 10 | 16 | 15 |
| #24 | 1 | 5 | 4 | 2 |
| #11 | 3 | 1 | 0 | 0 |
| #15 | 2 | 0 | 3 | 3 |
| #16 | 1 | 0 | 1 | 0 |
| #22 | 0 | 1 | 0 | 0 |

*Shadowed area: CS values, uninfected control animals; White area: CS values, inoculated animals. wpi: weeks post infection
